# Supplementary material for: Fully autonomous mouse behavioral and optogenetic experiments in home-cage
Source: eLife. 2021 May 4;10:e66112. doi: 10.7554/eLife.66112 (PMC8116056; doi:10.7554/eLife.66112)
Supplement: Supplementary file 1. [file elife-66112-supp1.docx]

$\sum_{i=1}^{20} S_{i}/20$ $\beta_{0}$

$$P\left( left \right)=\frac{1}{1+e^{-(z)}}$$

$$z=\beta_{s_{0}}S_{0}+\sum_{i=1}^{5} \beta_{s_{i}}S_{i}+\sum_{i=1}^{5} \beta_{A_{i}}A_{i}+\sum_{i=1}^{5} \beta_{R_{i}}R_{i}+\beta_{s_{avg.}}S_{avg.}+\beta_{WSLS}WSLS+\beta_{0}$$

$\beta_{0}$**Supplementary File 1.** Comparison with previous automated home-cage training systems with voluntary head-fixation.

| Features | Scott et al., 2013 | Murphy et al., 2016 | Aoki et al., 2017 | Murphy et al., 2020 | This study |
| --- | --- | --- | --- | --- | --- |
| Animal model | Rat | Mouse | Mouse | Mouse | Mouse |
| Housing | Rats are placed into test chambers in daily sessions | Home-cage testing; group housed | Singly housed mice accessed an external test chamber | Home-cage testing; group housed | Home-cage testing; singly housed |
| Head-fixation mechanism | Pistons press against headbar | Pistons press against headbar | Latching | Server motors press against headbar | Pistons press against headbar |
| Head-fixation stability | 1.6, 1.9 and 2.7 µm in x, y and z axis | 45, 45 and 9.5 µm in x, y and z axis | NA | NA | 6.4, 8.8 and 12.1 µm in x, y, and z axis |
| Head-fixation duration | 0.6 - 8 sec /fixation; Avg. 10 min/day | 30 sec /fixation; Avg. 6 min/day | 20-30 min per session | 19-45 sec /fixation; Avg. 18 min/day | 30-60 sec /fixation; Avg. 69 min/day |
| Self-release | Yes | No | No | No | Yes |
| Pre-training acclimation | No | Yes | Yes | Yes | No. |
| Number of days tested | Up to 140 days | Up to 90 days | Up to 84 days | 53 ± 32 days | 72 - 125 days |
| Number of trials /day in behavioral tasks | 110 trials/day for 7 s head-fixation    362 trials/day for 1.1 s head-fixation  510 trials/day for 0.6 s head-fixation | Not engaged in a behavioral task. | 120 trials/session | 100-200 trials/day | > 500 trials/day |
| Number of mice tested in automated system | 22 | 16 | 10 | 52 (28 good performers) | 65 |
